# Efficacy and safety of an NRTI-sparing regimen in antiretroviral-naïve HIV-infected patients: once-daily maraviroc plus lopinavir/ritonavir

**DOI:** 10.1186/1758-2652-13-S4-P5

**Published:** 2010-11-08

**Authors:** S Nozza, L Galli, M Di Pietro, F Mazzotta, F Canducci, M Pogliaghi, S Chiappetta, A Galli, V Rusconi, S Salpietro, G Tambussi, A Lazzarin

**Affiliations:** 1 San Raffaele Scientific Institute, Infectious Diseases, Milan, Italy; 2 H. S. M. Annunziata, Infectious Diseases, Antella, Florence, Italy

## 

Current guidelines recommend three drug combinations to treat antiretroviral naïve HIV-infected patients; some data of novel strategies with NRTI-sparing regimen in this setting are now available [1,2]. The study compares immunovirological efficacy and safety of once daily maraviroc (MVC) plus lopinavir/ritonavir (LPV/r) to tenofovir/emtricitabine (TDF/FTC) plus LPV/r.

This is an ongoing, proof-of-concept, randomized, open-label, 48 weeks trial. Data were collected at baseline (BL) and at 4, 12 , 24, 36 and 48 weeks. Comparisons between groups evaluated by the chi-square or Mann-Whitney rank-sum test. Results reported as median (Q1-Q3) or frequency (%), as appropriate.

Up to date, 13 pts (7 MVC, 6 TDF/FTC) reached week 24 (W24), 10 (5 MVC, 5 TDF/FTC) week 12 (W12), 1(MVC) week 4 (W4) without modification of the initial regimen; age 41.1 (41.7-49.9) years, 1/24 (4%) female, infected since 3.8 (2.3-7.6) years, CD4 nadir 266 (240-321) cells/µL. At BL: CD4 284 (260-325) cells/µL; CD4% 17.6 (13.9-22.5), HIV-RNA 4.3 (3.9-4.9) log_10_ copies/mL. No difference in BL characteristics were found between the two treatment groups.

At W24, all patients in both groups had HIV-RNA<50 copies/ml; decrease in viral load was similar in both groups at each timepoint. CD4 cells count increased in both groups, more rapidly in MVC group and was similar at week 12 and week 24, possibly due to the small number of subjects [CD4 change at W4: MVC group 183(134-225); TDF/FTC group 60(25-147), p=0.027; at W12: MVC group 161.5(141.5-216); TDF/FTC group 122(53.5-203), p=0.087; at W24: MVC group 174(72-179); TDF/FTC group 171(53.5-203), p=0.158].

Treatment was well tolerated, without grade 3 or 4 adverse events. No significant differences between the two groups were observed in bone marrow function, AST, ALT and CPK values, creatinine value, glucose profile (fasting glucose and insulin) and lipid profile (total cholesterol, LDL and HDL cholesterol) expect for triglycerides that significantly increased in TDF/FTC group [W24 increase in MVC group: 40 mg/dl (-3-299); TDF/FTC group: 119 (-29-137), p=0.037].

In this small sample size, NRTIs-sparing regimen with Maraviroc QD and lopinavir/ritonavir is similar in efficacy and tolerability to conventional treatment in naïve-patients. A more favourable trend in immunological recovery was observed but it needs to be confirmed in larger samples. Regimens NRTIs-sparing in HIV-infected patients naïve to antiretroviral therapy should be explored.
